# Screening tools to identify patients with complex health needs at risk of high use of health care services: A scoping review

**DOI:** 10.1371/journal.pone.0188663

**Published:** 2017-11-30

**Authors:** Valérie Marcoux, Maud-Christine Chouinard, Fatoumata Diadiou, Isabelle Dufour, Catherine Hudon

**Affiliations:** 1 Faculté de Médecine et des Sciences de la Santé de l’Université de Sherbrooke, Université de Sherbrooke, Québec, Canada; 2 Département des Sciences de la Santé, Université du Québec à Chicoutimi, Québec, Canada; 3 Centre de Recherche du Centre Intégré Universitaire de Santé et de Services Sociaux du Saguenay-Lac-Saint-Jean, Québec, Canada; 4 Centre de Recherche du Centre Hospitalier Universitaire de Sherbrooke, Québec, Canada; Universita degli Studi di Firenze, ITALY

## Abstract

**Background:**

Many people with chronic conditions have complex health needs often due to multiple chronic conditions, psychiatric comorbidities, psychosocial issues, or a combination of these factors. They are at high risk of frequent use of healthcare services. To offer these patients interventions adapted to their needs, it is crucial to be able to identify them early.

**Objective:**

The aim of this study was to find all existing screening tools that identify patients with complex health needs at risk of frequent use of healthcare services, and to highlight their principal characteristics. Our purpose was to find a short, valid screening tool to identify adult patients of all ages.

**Methods:**

A scoping review was performed on articles published between 1985 and July 2016, retrieved through a comprehensive search of the Scopus and CINAHL databases, following the methodological framework developed by Arksey and O’Malley (2005), and completed by Levac et al. (2010).

**Results:**

Of the 3,818 articles identified, 30 were included, presenting 14 different screening tools. Seven tools were self-reported. Five targeted adult patients, and nine geriatric patients. Two tools were designed for specific populations. Four can be completed in 15 minutes or less. Most screening tools target elderly persons. The INTERMED self-assessment (IM-SA) targets adults of all ages and can be completed in less than 15 minutes.

**Conclusion:**

Future research could evaluate its usefulness as a screening tool for identifying patients with complex needs at risk of becoming high users of healthcare services.

## Introduction

A number of people with chronic conditions require more services due to characteristics that increase their vulnerability, such as multiple chronic conditions, psychiatric comorbidities, psychosocial issues, or a combination of these factors.[1] They have to consult multiple healthcare and social services professionals, which increases the risk of care fragmentation and the frequency of use of healthcare services.[2, 3] Case management (CM) is recognized as an effective approach for improving the satisfaction and quality of life of patients with complex needs while reducing inappropriate use of healthcare services as well as costs.[4, 5]

Case finding consists in identifying patients with complex needs at risk of becoming high users of healthcare services, for whom a CM intervention could be beneficial.[6] In a systematic review documenting risk prediction models for emergency hospital admission for community-dwelling adults, the six risk prediction models that performed best included similar variables, namely, prior healthcare utilization, multimorbidity or polypharmacy measures, and named medical diagnoses or named prescribed medications predictor variables.[7] A scoping review performed to identify the predictive factors of frequent Emergency Department (ED) use indicated that people with low socioeconomic status, high levels of healthcare use (other than ED), and suffering from multiple physical and mental conditions were more likely to be frequent ED users.[8] To our knowledge, there are no reviews presenting and comparing various tools available to support case finding of patients with complex health needs at risk of frequent use of healthcare services.

The aim of this study was to find existing screening tools that identify patients with complex health needs at risk of frequent use of healthcare services and to highlight their principal characteristics. Our purpose was to find a short (less than 15 minutes), valid screening tool to identify adult patients of all ages.

## Materials and methods

### Protocol

We used the methodological framework for conducting a scoping review developed by Arksey and O’Malley (2005),[9] and completed by Levac et al. (2010),[10] to examine the extent and nature of research on the topic, [9]. Five steps were followed: 1) identifying the research question; 2) identifying relevant studies; 3) selecting studies; 4) charting the data; and 5) collating, summarizing, and reporting the results.[11]

#### 1) Identifying the research question

Our primary research question was defined as follows: Which questionnaires or screening tools exist to identify patients with complex health needs at risk of frequent use of healthcare services, and what are the principal characteristics of these instruments? Does a short, valid screening tool exist to identify adult patients of all ages?

#### 2) Identifying relevant studies

A search strategy was developed with an information specialist to conduct a comprehensive literature search in July 2016 in two databases, CINAHL and Scopus (which includes EMBASE and MEDLINE), for articles in English published between 1985 (year of the oldest article retrieved by the first preliminary search strategy) and 2016. The following keywords and Boolean operators were used to find studies of interest: (heavy user OR super user OR repeat user OR frequent attend OR frequent consult OR high attend OR high use OR repeat use OR frequent flyer OR heavy use OR case management) AND (case finding OR identification OR finding OR tracking OR key question OR risk assessment OR risk prediction OR screening) AND (health service OR hospital OR emergency department). The search returned 994 articles in CINAHL and 2,803 articles in Scopus, for a total of 3,797 articles.

#### 3) Selecting studies (Fig 1)

Through database searching, 3,797 articles were identified. Twenty-one articles were identified through references from identified articles and contact with first author of articles of interesting tools. After removal of duplicates, 3,184 articles were screened by title and abstract based on the inclusion and exclusion criteria, to exclude clearly non-eligible articles (VM). In case of uncertainty, the full articles were retrieved and read by a second team member as well (CH). To be included in the review, studies had to: 1) present a questionnaire or a clinical screening tool to identify patients with complex needs at risk of frequent use of healthcare services; and 2) concern an adult population (18 years old or older). Studies limited to psychiatric and pediatric populations as well as pregnant women were excluded. We also excluded studies reporting predictive modelling methods based on insurance claims, algorithms, program software, and mathematical models if they did not present a clinical screening tool.

**Fig 1:**
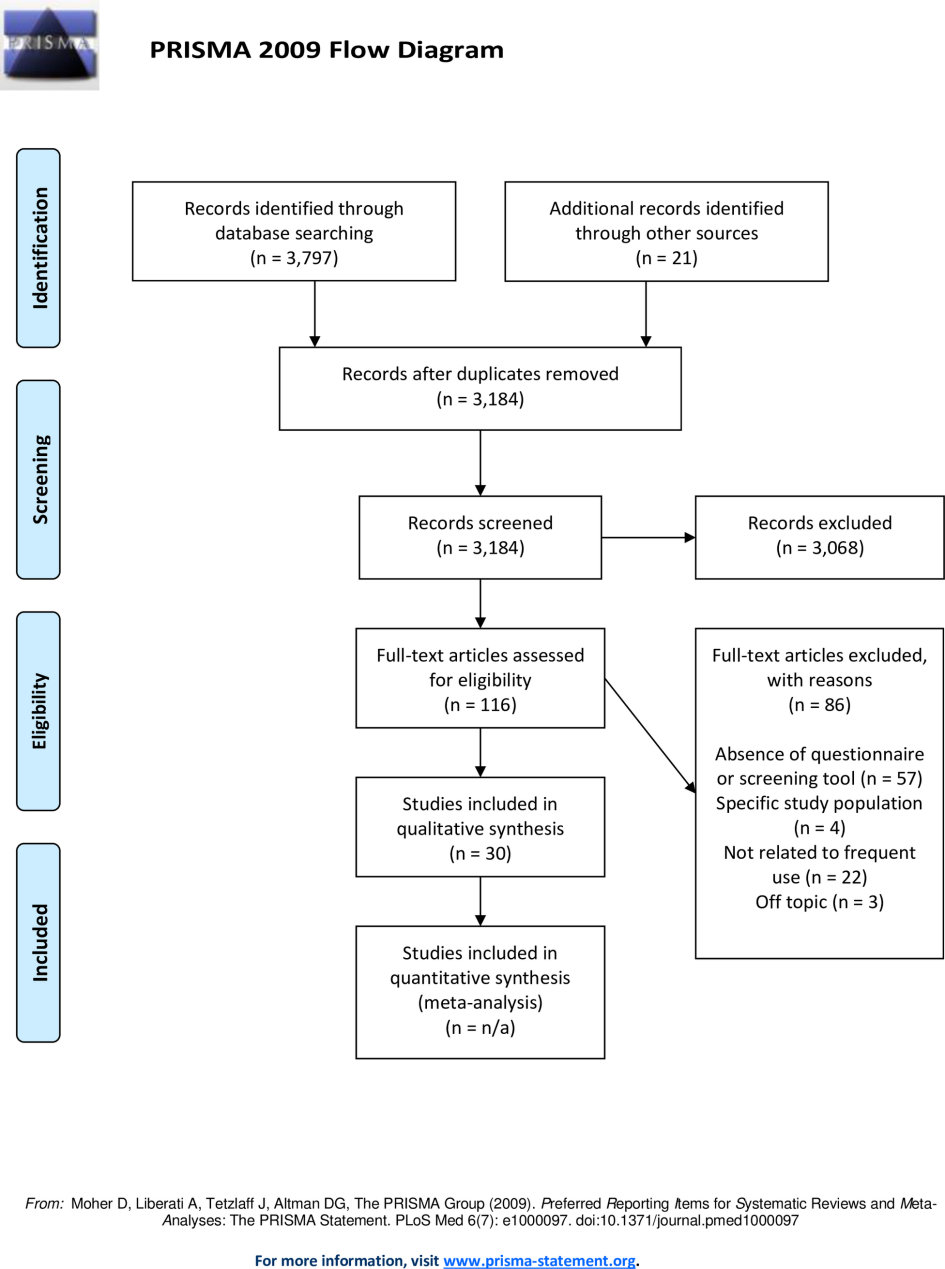
Scoping review–flowchart of search results.

One hundred sixteen articles were retained for detailed evaluation by two team members (VM and CH). Of these, 86 were excluded: 57 did not present a questionnaire or a screening tool; 4 were limited to a specific population; 22 were not related to frequent use of services; and finally, 3 were off topic. Thirty articles matched the inclusion criteria.

#### 4) Charting the data

Two authors (VM and FD) extracted the information from the 30 articles using an extraction grid. Conflicts were resolved by consensus. The names of authors, year, and country of development for each screening tool were based on the first publication about the instrument. We also extracted the population screened by the tool, the outcome (e.g. probability of a person being hospitalized within one year), the format (e.g. interview, self-reported information), the dimensions, the number of questions, and the time needed to complete.

Finally, we reported the development and validation steps of the tool, and the psychometric properties of the questionnaire (S1 Appendix).

#### 5) Collating, summarizing, and reporting the results

We collated, summarized, and reported the results using narrative synthesis.[12]

## Results

Thirty individual articles were included, encompassing 14 relevant questionnaires and screening tools (Table 1).

**Table 1:** Characteristics of questionnaires or screening tools.

Tool and references	Authors, year and country of first article about the tool	Characteristics of population screened by the tool	Outcome	Format of the tool	Dimensions or number of questions evaluated by the tool	Time needed
Probability of Repeated Admission (Pra)[13–22]	1. Boult, C. 2. Dowd, B. 3. McCaffrey, D. 4. Boult, L. 5. Hernandez, R. 6. Krulewitch, H. 1993, USA	Elderly persons 65 years or older	Probability of an elderly person being hospitalized within 4 years	Post or telephone interview	1. Availability of a caregiver 2. Diabetes 3. Age 4. History of coronary artery disease 5. Hospital admission during the past year 6. Gender 7. More than 6 physician visits during the past year 8. Poor self-rated general health Total: 8 questions	5 minutes
Triage Risk Screening Tool (TRST) [23–25]	7. Mion, L.C. 8. Palmer, R.M. 9. Anetzberger, G.J. 10. Meldon, S. W. 1997, USA	Community-dwelling elders (65 years and older)	EmergencyDept. (ED) revisits, hospitalizations, or long-term care placement within 30 and 120 days after an ED visit	Nurse screened all eligible patients	1. Cognitive impairment 2. Self-reported difficulty in walking or transferring 3. Use of 5 or more medications 4. ED visit within the previous 30 days or a hospital admission within the previous 90 days 5. ED Registry nurse recommendations Total: 5 questions	Not reported. Estimated: 5 minutes
“Initial assessment interview question”[20]	11. Boult, C. 12. Pualwan, T.F. 13. Fox, P.D. 14. Pacala, J.T. 1998, USA	Seniors	High-risk seniors likely to benefit from multidimensional intervention	Telephone or in-person interview	8 domains: 1. Cognition (4 questions) 2. Medical conditions (3 questions per health problem) 3. Medications (4 questions) 4. Access to care (8 questions) 5. Functional status (8 questions) 6. Social situation (3 questions) 7. Nutrition (minimum 1 question if no weight change, maximum of 3 questions) 8. Emotional status (2 questions) Minimum number of questions: 33; Maximal number of questions: relative to the number of health problems and change of weight	30–45 minutes
INTERMED [26–33]	15. Huyse, FJ.1 16. Lyons, JS. 17. Stiefel, FC. 18. Slaets, JP. 19. de Jonge, P. 20. Fink, P. 21. Gans, RO. 22. Guex, P. 23. Herzog, T. 24. Lobo, A. 25. Smith, GC. 26. van Schijndel, RS. 1999 *, Netherlands *	(No age specifications)	Indicates bio-psychosocial health-care needs. The higher the score the higher the healthcare needs *	Observer-rated instrument	1. Biological: Chronicity and diagnostic uncertainty (history)/Severity of illness and clarity of diagnostic profile (current state) /Complications and life threat (short- and long-term) (prognosis) 2. Psychological: Restrictions in coping, premorbid level of psychiatric dysfunctioning (history)\Treatment resistance, severity of psychiatric symptoms (current state)\ Mental health threat (short- and long-term) (prognosis) 3. Social: Family disruption, impairment of social support (history) \Residential instability, impairment of social integration (current state)\Social vulnerability (short- and long-term) (prognosis) 4. Healthcare system: Intensity of prior treatment, prior treatment experience (history)\ Organizational complexity at admission or referral, appropriateness of admission or referral (current state) \Care needs (short- and long-term) (prognosis) Total: 20 questions [30]	20–30 minutes (at least) *
Community Assessment Risk Screen (CARS)[19, 34]	5. Shelton, P. 6. Sager, M.A. 7. Schreaeder, C. 2000, USA	Patients 65 years and older	Patients at risk of hospitalization or having an ED encounter during the subsequent 12 months [34]	Questionnaire filled out by medical staff through an interview or filled by post	1. Preexisting chronic diseases 2 or more (heart disease, diabetes, myocardial infarction, stroke, chronic obstructive pulmonary disease, cancer) 2. Prescription for 5 or more medications 3. Hospitalization or ED use in the preceding 6 months Total: 3 questions[34]	Not reported. Estimated: less than 5 minutes
Analysis of risk element/origin/resources/action(ARORA)[15]	8. Smith, T. 2000, USA	Geriatric patients	Individuals at risk for hospitalization[15]	Two phases: 1) Worksheet completed by medical staff. 2) Algorithm that provides a systematic approach for intervention	7 risk elements: 1. Cognitive function 2. Medical conditions 3. Medications 4. Care access 5. Functional status 6. Nutrition 7. Emotional status For each risk element, there are these 6 questions to answer: 1. Adequate access to medical care 2. Coordination of medical care 3. Patient awareness of all medical needs 4. Patient awareness of self-care needs 5. Presence of mechanical or physical hazards 6. Adequate assistance for self-care Total: 42 questions	Not reported. Estimated: more than 30 minutes
Health Perception Assessment (HPA) Instrument, later named One Care Street Health Profile [35]	- Meek, J. A. - Lyon, B. L. - May, F. E. - Lynch, W. D. 2000, USA	Non-institutionalized enrollees of the health plan, ranging in age from 18 to 65 years old	High use of the healthcare system over the next 6 months	Self-reported mailed questionnaire	1. Physical symptoms 2. Levels of emotions 3. Levels of functioning 4. Beliefs / preferences regarding the healthcare system 5. Health risk 6. Disease 7. Compliance 8. Demographic items (The number of questions per dimension evaluated was not mentioned) Initial version: 70 questions with 126 responses. Shortened version after trial of the predictive model: 48 questions with 74 responses	Not reported. Estimated: more than 30 minutes
Reuben et al. [16]	- Reuben, D. B. - Keeler, E. - Seeman, T. E. - Sewall, A. - Hirsch, S. H. - Guralnik, J. M. 2002, USA	Beneficiaries aged 71 years and older	Patients at high risk for high health care utilization (defined as ≥11 hospital days over 3 years)	Two phases: 1) Self-report by mail, telephone, or possibly, Internet survey. 2) Laboratory tests	First phase: (4 of the 8 Pra questions). 1. Hospitalizations in previous year 2. Hospitalizations in last two years 3. Gender 4. Self-reported health 5. Employment 6. Participation at religious services 7. Need help with bathing 8. Mobility (unable to walk ½ mile) 9. Diabetes 10. Medication (loop diuretic) Second phase: 2 laboratory tests 1. Serum albumin (<3.8 g/dL) 2. Serum iron (<48 mcg/dL) Total: 10 questions and 2 laboratory tests	Not reported. Estimated: 10–15 minutes (without laboratory tests)
Annual screening questionnaire [36]	- Graves, T. - Slater, M.A. - Maravilla, V. - Reissler,L. - Faculjak, P. - Newcomer, R.J. 2003, USA	80 years and older	Overall risk score: high priority, medium, or low	Mailed questionnaire	1. Cognitive/affective 2. Disease 3. Financial/legal 4. Functional status 5. Home environment/safety 6. Lifestyle 7. Medication 8. Nutrition 9. Social/cultural 10. Services utilization (number of questions per domains not mentioned) Total: 85 questions	30–45 minutes
Predicted Insurance Expenditures (Pie) [37]	- Boult, C. - Kessler, J. - Urdangarin, C. - Boult, L. - Yedidia, P. 2004, USA	Full-time adult (21–64 years old) employees with insurance coverage	Heavy users of healthcare services	Short self-report survey scored electronically	Two demographic variables: 1. Advanced age (50 years old and more) 2. Gender Five medical variables: 1. Arthritis causing pain most days 2. High cholesterol 3. Diabetes 4. Cancer 5. Regular use of medication One use-of-services variable: 6. Hospitalization more than 1 time in the previous year 7. Clinic/physician visits more than 3 times in previous year 8. ED visit more than 1 time in previous year Clinic or physician visits more than 1 time during the previous year Total: 8 questions	1–2 minutes
INTERMED for the Elderly (IM-E) [26, 38]	- Söllner, W. - Wild, B. - Lechner, S. - Holzapfel, N. - Slaets, J. - Stiefel, F. - Huyse, F. * 2008 *, Germany*	Elderly patients	Bio-psychosocial healthcare needs*	A highly structured interview* and scoring guide that was recorded in a standardized blank form	(modifications of INTERMED) 1. Biological - Chronicity and diagnostic dilemma (history) - Severity of symptoms, diagnostic problems (current state) - Complications and life threat (prognosis) 2. Psychological - Restrictions in coping psychiatric dysfunctioning (history) - Treatment resistance, psychiatric symptoms (current state) - Mental health threat (prognosis) 3. Social - Restriction in social integration, social dysfunctioning (history) - Residential instability (current state) - Social vulnerability (prognosis) 4. Healthcare system - Intensity of treatment, treatment experience (history) - Complexity of care, appropriateness of care (current state) Coordination of care (prognosis) Total: 20 questions	20–30 minutes (at least)*
INTERMED Self-Assessment (IM-SA) [39]	- Slaets, J. - Stiefel, F. - Ferrari, S. - Huyse, F. - Latour, C. - Boenink, A. - Söllner, W. - Wild, B. * 2008–2010, developed in English; translated into Italian, Spanish, French, Dutch, German *, The Netherlands*	Various patient populations	Bio-psychosocial healthcare needs and the higher the score the higher the healthcare needs	Self-assessment questionnaire	26 questions to be assessed regarding four dimensions: 1. Biological 2. Psychological 3. Social 4. Healthcare use (same domains evaluated as INTERMED original version)	10–12 minutes*
Homeless Screening Risk of Re-Presentation [40]	- Moore, G. - Hepworth, G. - Weiland, T. - Manias, E. - Gerdtz, M.F. - Kehaler, M. - Dunt, D. 2012, Australia	Homeless people who went to the ED	Re-presentation to the ED within 28 days of discharge from hospital	Risk screening tool administered by clerical staff upon presentation to the ED	1. Age group 2. Known next of kin 3. Pensioner (yes/no) 4. Primary presenting problem (medical/surgical, injury, mental illness, drug and alcohol) 5. Number of medical issues (3 or less, 4 to 7, 10 to 14, 15 and more) 6. Number of medications prescribed (4 or less, 5 to 9, 10 to 14, 15 or more) 7. Community support 8. Presented to other hospitals within the last 12 months 9. Discharge outcomes (admit, return to ward, home, ED admit, transferred, left before treatment, residential care facilities) Total: 9 questions	Not reported. Estimated: 5 minutes
INTERMED for the Elderly Self Assessment (IM-E-SA)* [41, 42]	- Peters, L.L. - Boter, H. - Slaets, J.P.J. - Buskens, E.* 2013*, The Netherlands*	Patients 65 years of age and older	Curative care costs in the follow-up year[41]	Self-assessment version [42]	Same as IM-E: the IM-E-SA assesses case complexity and healthcare needs in the following domains: 1. Biological 2. Psychological 3. Social 4. Healthcare All domains comprise five questions, with each domain begin assessed in the context of time (history, current state, and prognosis). Total: 20 questions	15 minutes *

* Information obtained by contacting authors.

### Study characteristics

Seven studies were published between 2010 and 2016,[19, 26, 38–42] 16 were published between 2000 and 2009,[13–16, 18, 23–25, 29, 31–37] and 7 were published between 1990 and 1999.[17, 20–22, 27, 28, 30]

Fourteen studies were from the USA,[14–18, 20–24, 34–37] 5 were from the Netherlands, [30, 32, 33, 41, 42] 5 were from Switzerland,[13, 27–29, 31] 4 were from Germany,[13, 26, 38, 39] and 4 other studies were from Spain,[19] United Kingdom,[13] Canada,[25] and Australia,[40] respectively. One study on a self-administered questionnaire was conducted in three European countries—Germany, United Kingdom, and Switzerland.[13]

Fourteen articles presented the development of the questionnaire or the screening tool, [15, 16, 21–23, 26, 30, 34–37, 39, 40, 42] while 16 others were validation studies.[13, 14, 17–20, 24, 25, 27–29, 31–33, 38, 41]

### Type of questionnaires

Seven questionnaires were self-reported (Pra, HPA, Reuben et al., Annual screening questionnaire, Pie, IM-SA, IM-E-SA), 7 were conducted by an interviewer (Initial assessment interview question, INTERMED, CARS, ARORA, TRST, IM-E, Homeless Screening Risk of Re-Presentation), and 1 tool used laboratory tests (Reuben et al.). Two tools had 5 questions or less (CARS, TRST), 4 tools had 5 to 10 questions (Pra, Reuben et al., Pie, Homeless Screening Risk of Re-Presentation), 3 tools had 10 to 20 questions (INTERMED, IM-E, IM-E-SA), and 5 tools had more than 20 questions (HPA, Initial assessment interview question, ARORA, annual screening questionnaire, IM-SA).

Of the instruments reporting the time needed to complete, 2 screening tools reportedly took 5 minutes or less (Pra, Pie), 2 tools took 10 to 15 minutes (IM-SA, IM-E-SA), 2 tools took 20 to 30 minutes (INTERMED, IM-E), and 2 tools took 30 to 45 minutes (Initial assessment interview question, annual screening questionnaire). In short, 4 tools were reported as requiring less than 15 minutes to use.

### Population aimed

Five instruments were intended for adult patients of all ages (INTERMED, IM-SA, Homeless Screening Risk of Re-Presentation, HPA, Pie), and 9 were intended for geriatric patients (“Initial assessment interview question”, ARORA, annual screening questionnaire, IM-E, IM-E-SA, Pra, CARS, Reuben et al., TRST). Two tools were designed for specific populations: the Homeless Screening Risk of Re-Presentation for homeless people, and the Pie tool for new adult employees.

### Prediction outcomes

Three tools (Pra, CARS and TRST) predict the probability of being hospitalized within a set period of time. Otherwise, most of the tools predict higher use of healthcare services, while a few are used, for example, to calculate an overall risk score or predict curative care cost in the follow-up year.

### Development and validation steps

In the cases of 5 of the included screening tools, their authors used multivariate logistic regression to identify which predictors were significant enough to be included in their questionnaire or tool. The authors of the INTERMED questionnaire used an existing bio-psychosocial model to create their management tool. It was later modified to produce a version focusing on older persons, the INTERMED for the Elderly (IM-E). Subsequently, self-assessment versions of the INTERMED (IM-SA) and the IM-E (IM-E-SA) were developed, mostly by rephrasing the questions to improve clarity. Many of these tools have also been translated into many languages, such as Italian, Dutch, and German, to facilitate their use in other countries. Table 2 presents best predictors included in the questionnaires. Prior healthcare utilization (n = 9), medical conditions (n = 10) and medications (n = 8) were the most frequently used predictors in the questionnaires, while emotional status (n = 6) and socioeconomic condition (n = 5) were less frequently used.

**Table 2:** Best predictors included in the questionnaire or screening tools [7–8].

Instrument	Prior healthcare utilization	Medical conditions (self-reported) medical diagnosis	Medications	Emotional status /mental health	Socioeconomic condition
Probability of Repeated admission (Pra)	x	x			
Triage Risk Screening Tool (TRST)	x		x		
«Initial assessment interview question»	x	x	x	x	x
INTERMED, IM-SA, IM-E, IM-E-SA	x	x		x	x
Community Assessment Risk (CARS)	x	x	x		
Analysis of risk element/origin/ resources/action (ARORA)		x	x	x	
Health Perception Assessment (HPA)		x		x	
Reuben et al.	x	x	x		x
Annual screening questionnaire	x	x	x	x	x
Predicted Insurance Expenditures (Pie)	x	x	x		
Homeless Screening Risk of Re-Presentation	x	x	x	x	x

### Psychometric properties

Six screening tools have validation studies in other contexts. Results concerning the psychometric properties of the instruments under study are presented in S1 Appendix. The most strongly validated tools are the Pra and the INTERMED.

## Discussion

The purpose of our study was to describe all existing screening tools that identify patients at risk of frequent use of healthcare services, in order to find a short, valid screening tool for identifying adults of all ages. Of the 14 instruments presented, 5 screened an adult population (18 years and older): INTERMED, IM-SA, Homeless Screening Risk of Re-Presentation, HPA, and Pie. INTERMED is a reliable and well-documented validated assessment tool integrating biological, psychological, social, and healthcare domains, but it must be observer-rated and takes between 20 and 30 minutes. IM-SA is a reliable self-administered instrument that targets adult of all ages and can be completed in less than 15 minutes, the only one corresponding to our criteria. Homeless Screening Risk of Re-Presentation is designed to predict healthcare services use of the homeless only. The HPA is a relevant self-reported instrument predicting high use of the healthcare system, but is very long to administer (48 questions). Pie is a self-reported survey that is scored electronically, takes about one to two minutes to complete, and can identify high potential users of healthcare services for adults (21–64 years), but it is mainly intended to predict heavy use of healthcare services by new employees only.

To our knowledge, this is the first review in which available screening tools identifying adult patients of all ages at risk of frequent use of healthcare services have been presented and compared. A systematic review published in 2014 focused on risk prediction models for emergency hospital admission screening populations aged 18 years old and over.[7] That review discussed 3 screening tools: the tool presented by Reuben and al., the Pra, and the CARS. Our scoping review includes 11 other instruments. Another systematic review looked at questionnaires identifying seniors (50 years and older) living in the community who were at risk of hospitalization, loss of autonomy, institutionalization, or death.[43] They also included the tool presented by Reuben and al., the Pra, and the CARS. Another one aimed to quantify the prognostic accuracy of individual risk factors and ED- validated screening instruments to distinguish patients of 65 years and older at risk of emergency department returns, hospital readmissions, functional decline, or death.[44] They also included the TRST. The current scoping review added at least 11 new tools not included in these systematic reviews.

We used a strong research strategy developed in collaboration with an information specialist to enhance our comprehensive database search. All articles were reviewed by two independent authors to reduce the risk of selection bias. Moreover, the first author (VM) attempted to contact all first authors of articles that presented the development of a questionnaire or screening tool. Five authors replied, providing additional information or literature about their tool.

One limitation is that we have not conducted the optional sixth step of a scoping review, which is consultation with stakeholders to include supplementary sources of information, perspectives, and applicability.[10] Another limitation is that a scoping review is not meant to evaluate the quality of the articles included. Finally, the potential omission of relevant articles, as well as any unpublished material, is also a limitation of this study.

## Conclusion

Most screening tools target elderly persons. IM-SA targets adult of all ages and can be completed in less than 15 minutes. Future research could evaluate its usefulness as a screening tool for identifying patients with complex needs at risk of becoming high users of healthcare services, for whom a CM intervention could be beneficial.

## Supporting information

S1 Appendix (DOCX)

S1 Table (DOC)
