# Supplementary material for: Screening tools to identify patients with complex health needs at risk of high use of health care services: A scoping review
Source: PLoS One. 2017 Nov 30;12(11):e0188663. doi: 10.1371/journal.pone.0188663 (PMC5708762; doi:10.1371/journal.pone.0188663)
Supplement: S1 Appendix — (DOCX) [file pone.0188663.s001.docx]

**S1 Table. Development steps and psychometric properties of questionnaires or screening tools**

| **Tool and references** | **Tool development steps** | **Tool validation steps** | **Metrological properties of the tool according to validation studies** |
| --- | --- | --- | --- |
| Probability  of Repeated  Admission (Pra)[13, 21-25, 36-39] | 1. Evaluation of six predisposing variables, one enabling variable and 21 need variables as possible predictors of repeated hospital admission.  2. First half of the sample was fitted to an initial multivariate logistic regression model of predictors of repeated admissions  3. Possible interactive effects were considered for those variables whose initial regression coefficients had significance levels of less than 0.10  4. Evaluation of pairs of variables for which interactions seemed plausible using two-by-two contingency tables and the Breslow-Day test for homogeneity of odds ratios.  5. Expansion of the logistic model to include the interactions that appeared to be significant in the contingency tables.  6. Variables whose final logistic regression coefficients had significance levels of less than 0.10 were selected as screening criteria for high risk of repeated admission | 1. Calculate with the final logistic model the probability of repeated admission for each subject in the second half of the sample.  2. Predict the cut-off score to identify high-risk patients.  3. Test the prediction with a comparison of the observed experiences of the high-risk subjects to those of the low-risk subjects.  4. Repeat all of these regression and validation studies using three alternate definitions of repeated admission to evaluate the sensitivity of the results to the initial definition. | A retrospective study in Spain found that the CARS and Pra tools predict with high efficacy the proportion of patients not readmitted (negative predictive value between 91% and 92%).  Pra’s sensitivity: 54%  Pra’s specificity: 81% [13] |
| Triage Risk Screening Tool (TRST) [26-28] | 1. An expert panel of physicians, all specializing in gerontology, reviewed the multiple risk factors for clinical applicability and feasibility in the Emergency Department (ED) setting.  2. Five risk factors were chosen for inclusion in the initial screening instrument, which had been tested in a 2-week pilot study at two of the ED sites.  3. An additional factor was added on the recommendation of ED nursing personnel, termed ‘professional recommendation’, which reflects the ED nurse’s clinical judgment. | 1. The TRST variables, treated dichotomously, were entered into a logistic regression model. Separate models for 30- and 120-day outcomes were derived.  2. Bayesian information criteria (BIC) comparison techniques were used to compare models.  3. Receiver operating character characteristic (ROC) curves were constructed, and the area under the curve (AUC) was calculated.  4. Predictive value of the TRST for the composite endpoint and individual healthcare utilization was examined by calculating relative risks of these outcome measures.  5. 30- and 120-day outcomes were examined using chi-square tests for categorical variables, Wilcoxon rank sum for non-normally distributed variables, and t-tests for continuous variables.[28] | Patients defined as high risk by the TRST were significantly more likely to require subsequent ED use (RR=1.7; 95% CI = 1.2 to 2.3), hospital admission (RR=3.3; 95% CI=2.2 to 5.1), or the composite outcome (RR=1.9; 95% CI 1.7 to 2.9) at both 30 days and 120 days than the low-risk cohort.  The TRST with a cut-off score of 2 was the most parsimonious model for predicting composite outcomes (AUC = 0.64) and hospitalization at 30 days (AUC = 0.72).[28]  A Canadian study[26] demonstrates that the TRST is a poor diagnostic test to predict ED revisit, hospital readmission, or long-term care placements at 30 and 120 days by the failure of the likelihood ratios CIs to achieve levels of clinical significance. |
| Initial assessment interview question[36] | - | - | - |
| INTERMED [14, 29-32, 40-42] | 1. Combination of a biopsychosocial grid and measurement strategies to create a case-mix, decision-support and outcome-management tool.  2. The data has been synthesized for four systems (biological, psychological, social, healthcare) and assessed in the context of time (history, current state and prognosis).  3. Within each of the resulting domains, two pertinent variables were chosen on clinical and/or scientific grounds known to be important for each of the domains, representing an indicator for the past, current, or future needs in this domain.  4. Each of the variables of the different domains have to be scored according to a defined scoring system, ranging from a score of 0 (no vulnerability or need) to a score of 3 (high vulnerability or need) | 1. A first study on the instrument was conducted, in which the INTERMED was double scored for 14 patients admitted to an internal ward by a psychiatrist and an internist on the basis of a joint interview conducted by both. The results were analyzed, which led to improvements of the instrument.  2. A second study was performed in which two clinicians separately double scored the INTERMED for 16 patients referred to the outpatient psychiatric consultation service, on the basis of medical charts. The results of this study were analyzed and led to the definitive version of the INTERMED. | All Cronbach’s alpha’s were above .75, indicating satisfactory internal consistency in each of the samples. The Cronbach’s alpha of the total sample was .87 and between .86 and .89 with a 95% level of confidence interval.[31]  Correlations between total scores from two different raters ranged from 0.91–0.96.[30] |
| Community Assessment Risk Screen (CARS)[13, 33] | 1. Implementation of a prospective cohort study.  2. Baseline demographic, health status, and utilization measures were obtained from telephone interviews and mailed questionnaires. Service utilization data were obtained from Medicare claims files.  3. Utilization and cost data for the validation cohort were obtained from submitted claims.  4. Logistic regression was used to identify 3 factors that predicted a hospitalization or ED visit during the first year of the study.  5. A scoring system (from 0 to 9) was developed for each predictor variable. | 1. Patients in the validation cohort were assigned to low (0 to 3) and high (4 to 9) risk categories.  2. Comparison of the low-risk and the high-risk groups. [33] | CARS demonstrated a good degree of predictive discrimination as indicated by the area under the ROC curve of 0.67 when a cut-off score of 4 or more was used to categorize high-risk patients in the validation cohort.[33]  A retrospective study in Spain found that the CARS and Pra tools predict with high efficacy the proportion of patients not readmitted (negative predictive value between 91% and 92%).  CARS’s sensitivity: 64%  CARS’s specificity: 64% [13] |
| Analysis of risk element/origin/resources/ action (ARORA)[22] | - | - | - |
| Health Perception Assessment (HPA) instrument, later named One Care Street Health Profile[20] | 1. Logistic regression  2. Conversion of the resulting model to a formula yielding a probability value  3. Subsequent testing of the formula using Chi Square on the first split-half population |  | Results showed that compared with the traditional method of identifying high-risk members (stratification methods, e.g. using past hospitalization, age and presence of chronic disease), the HPA added 6.6% to the sensitivity and 12.3% to the specificity in the first split-half, thus contributing considerable predictive power.  At the probability threshold value of 0.56:  HPA’s sensitivity: 66.7%  HPA’s specificity: 63.4%  Traditional method sensitivity: 60.1%  Traditional method specificity: 51.1% |
| Reuben et al.[21] | 1. Examination of possible risk-prediction variables, including self-report items, physical and cognitive examination findings, and laboratory values.  2. Selection of variables previously linked to subsequent healthcare utilization, functional decline, or mortality.  3. Analysis of the variables as continuous and categorical data according to thresholds that are commonly used in clinical practice.  4. Test the final models on subsample of subjects. | 1. Based on bivariate models between candidate variables and utilization outcomes; enter self-report items into stepwise multivariable models to create a self-report model.  2. Follow the same process with physical examination findings and laboratory items to create a combined self-report and physical examination and laboratory test model.  3. Test the hypothesis of the two-phase strategy.  4. Evaluate the possibility of a bias associated with using the same data for generating coefficients and estimating probabilities.  5. Examine the influence of mortality on utilization.  6. Compare the accuracy of the model with the Pra tool. | Approximately ¼ of the population was identified as being at high risk (≥0.28 probability) for high healthcare utilization and those identified accounted for approximately half of all Medicare Part A cost for the entire population.  The area under the receiver operating characteristic (ROC) curve was 0.68 for Phase 1. |
| PCSH’s 2000 mail questionnaire [34] | - | - | - |
| Predicted Insurance Expenditures (Pie) [35] | 1. Mail the 53-item Good Health Survey, a health risk appraisal (HRA) instrument to members of a “derivation cohort”.  2. Obtain records of the eligible respondents’ health insurance expenditures during the following year.  3. Using multiple linear regressions, identify 8 of the questions that predicted future expenditures most accurately, and created a formula to predict total expenditures from answers to these questions. | 1. Use the 8-item questionnaire to survey two validation cohorts.  2. Insert responses into the scoring formula.  3. Classify respondents into high-risk (top 10%) or low-risk (lower 90%) groupings.  4. Compare health insurance expenditures generated by the high- and low-risk groups during the following year. | The Pie scoring formula identified "high-risk" respondents (10%) who subsequently generated healthcare expenditures 2.4 and 1.8 times greater than those generated by “low-risk” respondents. |
| INTERMED for the Elderly (IM-E) [14, 15] | 1. In focus groups conducted in conjunction with the international authors of the original INTERMED, the variables, lead questions, anchor points, and scorings that had to be adjusted to the needs and situations of the elderly were discussed.  2. A first version of the IM-E was developed and approved by all the participants in the study group of the INTERMED Foundation.  3. Pilot testing of the new version was done with six patients. In addition, training and focus groups with medical doctors working in the ESTHER study were held.  4. Design of a short, clear, and practical interview structure and scoring procedure. A second round of training and focus group with different study doctors was conducted using videos of patient interviews and ratings according to the final version of the IM-E. | 1. Participants were interviewed by one of the two raters in the presence of the other and were subsequently independently doubly scored.  2. The interrater reliability of the four domain scores and the total score were calculated using the intraclass correlation (ICC) coefficient (2, 1) according to Shrout and Fleiss.  3. To assess agreement between the two raters regarding the dichotomizing cut-off point of 20, we calculated the overall percentage of agreement.  4. Internal consistency was determined by calculating Cronbach's α. Good internal consistency (.7≤α≤.9) can be seen as a precondition for considering that the summation of the single item scores to a total score is meaningful.[14] | The interrater reliability of the four domain scores and the total score were calculated using the intraclass correlation (ICC) coefficient (2, 1). ICCs for the various domains of the IM-E ranged between 0.87 and 0.95, while the ICC for the sum score was 0.95.  Regarding the cut-off point of 20/21, a κ of 0.75 was achieved.  Internal consistency was determined by calculating Cronbach’s alpha. For the two raters, the Cronbach’s alpha values were 0.87 and 0.83, indicating good internal consistency. |
| INTERMED Self-Assessment (IM-SA)[16] | Development of a self-administered version of INTERMEN | 1. Evaluation of Cronbach’s alpha.  2. Intra-class correlation (ICC) between IM-SA and INTERMED.  3. Convergent validity with quality of life (SF-36 and EQ-5D), mental health (Hospital Amxiety and Depression Scale - HADS), multimorbidity (Cumulative Illness Rating Scale - CIRS).  4. Predictive validity for healthcare use (self-reported number of visits to an emergency room, days of hospital admissions, outpatient contacts with specialists). | Cronbach’s alpha=0.80  ICC: -.78 (CI, 0.75-0.81)  Correlation with SF-36= -0.65  Correlation with EQ-5D= -0.59  Correlation with HADS=0.15  Correlation with CIRS=0.28  Good predictive validity for healthcare use at 3 and 6 months |
| Homeless Screening Risk of Re-Presentation[17] | 1. A model was fitted for each potential explanatory variable from a predictive model that identified risk factors associated with increase odds of representation to the ED.  2. Variables with a *P*-value < 0.05 were entered into backwards stepwise procedures.  3. Key variables identified as significant in the logistic regression model were converted into a scoring system utilized in the final risk screening tool.  4. Identification of the optimal cut-off score by comparing sensitivity, specificity, positive likelihood ratio (PLR), negative likelihood ration (NLR), and the accompanying ROC curve. | 1. A prospective cohort study was conducted over one month including all ED visits involving people classified as homeless.  2. Identification of homeless at high risk with the screening tool. Forms were labelled, dated, and given to ALERT who was blinded to the outcome measure of re-presentations.  3. The number of ED re-representations within 28 days was subsequently collected by the researcher.  4. Comparison of the current homeless population with the homeless population that spanned 2 years (2003/2004) and that was used to develop the risk screening tool. | Sensitivity: 98% (CI,0.92-0.99)  Specificity: 66% (CI, 0.57-0.74)  Positive predictive value: 68%  Negative predictive value: 98%  Positive likelihood ratio: 2.9 (CI, 2.2-3.7)  Negative likelihood ratio: 0.03 (CI, 0.01- 0.13) |
| INTERMED for the Elderly Self Assessment  (IM-E-SA) [23, 24] | 1. The items of the IM-E were rephrased to improve its clarity.  2. The item ‘chronicity’ of the biological domain was changed from one item into two items to differentiate between physical dysfunction and the occurrence of chronic diseases. The scores of both items are recoded back to one item.  3. In the healthcare domain, the focus of the item ‘complexity of care’ was changed from assessing the number of healthcare professionals involved to an evaluation of the collaboration between several healthcare professionals.[19] | 1. Participants were mailed a questionnaire and were involved in a semi-structured interview.  3. Baseline characteristics were analyzed using descriptive statistics.  4. The reliability of the IM-E-SA was analyzed by calculating the Cronbach α.  5. For domains scores and total scores, interrater agreement (IM-E-SA versus IM-E) was calculated with intraclass correlation coefficients.  6. Spearman rank correlations were calculated to assess the convergent and discriminant validity.  7. Post-hoc analyses were performed to assess psychometric properties of the IM-E-SA in specific subgroups and by using other cut-offs for the number of missing values on the IM-E-SA to calculate the total IM-E-SA score.  8. Post-hoc univariate and multivariate linear regression analyses were performed to assess whether demographic characteristics and/or measurement scores were associated with the differences between the IM-E-SA and IM-E scores.[19] | The reliability of the IM-E-SA was expressed as a Cronbach’s alpha of 0.78.  The intraclass correlation coefficients (ICCs) between the total scores of the IM-E-SA and the IM-E was 0.68.  Correlations evaluating the convergent validity were moderate to strong (0.50–0.70). The correlations assessing discriminant validity were moderate (0.38–0.53). [19]  The predictive validity for healthcare costs was evaluated.[18]  Adjusted β for IM-E-SA was 0.06 (95% CI 0.04-0.07). Case complexity was a significant predictor of curative care costs (adjusted β 0.03 [95 IC 0.02-0.05]). |
